# The Influence of Text Genre on Eye Movement Patterns During Reading

**DOI:** 10.3390/jemr18060060

**Published:** 2025-11-03

**Authors:** Maksim Markevich, Anastasiia Streltsova

**Affiliations:** Center for Cognitive Sciences, Sirius University of Science and Technology, Krasnodar Region, 354340 Sirius, Russia; streltsova.av@talantiuspeh.ru

**Keywords:** reading, eye tracking, scanpath analysis, text genre, text comprehension

## Abstract

Successful reading comprehension depends on many factors, including text genre. Eye-tracking studies indicate that genre shapes eye movement patterns at a local level. Although the reading of expository and narrative texts by adolescents has been described in the literature, the reading of poetry by adolescents remains understudied. In this study, we used scanpath analysis to examine how genre and comprehension level influence global eye movement strategies in adolescents (N = 44). Thus, the novelty of this study lies in the use of scanpath analysis to measure global eye movement strategies employed by adolescents while reading narrative, expository, and poetic texts. Two distinct reading patterns emerged: a forward reading pattern (linear progression) and a regressive reading pattern (frequent lookbacks). Readers tended to use regressive patterns more often with expository and poetic texts, while forward patterns were more common with a narrative text. Comprehension level also played a significant role, with readers with a higher level of comprehension relying more on regressive patterns for expository and poetic texts. The results of this experiment suggest that scanpaths effectively capture genre-driven differences in reading strategies, underscoring how genre expectations may shape visual processing during reading.

## 1. Introduction

Reading comprehension is a crucial skill that affects daily life, education, and work. At school, much of the knowledge is acquired by children and adolescents through reading. To be successful academically, it is important for them not only to understand various texts but also learn from these texts [1].

Being a complex skill, the level of reading comprehension is defined not only by the products of comprehension, i.e., indicators of what the reader has understood after reading is completed, but also by the processes of comprehension, i.e., cognitive activities by which the reader can comprehend the meaning of the given text [2]. Therefore, reading comprehension can be characterized as the sum of the cognitive processes that allow the reader to understand the text meaning, and the mental representations that are the products of these processes [3,4,5]. Despite the variation in structures of different reading comprehension models, most of them include lower-level processes associated with basic language skills (e.g., decoding) and higher-level processes associated with cognitive skills needed for discourse-level processing (e.g., inferencing) [3].

Research shows that successful readers tend to employ different cognitive and interpretive strategies based on the type of text they are reading and the type of task they are to complete based on the text [6,7]. Moreover, successful readers are able to draw conclusions from the text, use prior knowledge, and metacognitive skills while reading [8]. Thus, reading comprehension depends not only on the level of skills that are part of its structure, but also on related skills, which makes mastering reading comprehension more difficult for beginning and poor readers.

### 1.1. The Influence of Text Genre on Text Processing

At school, children and teenagers interact with texts of different genres. Traditionally, genre is defined as a class of communicative events that share some set of communicative purposes [9]. Although most educational materials are represented by expository texts, or informational texts, school subjects involving learning of a native or foreign language, literature, etc., which are crucial for a child’s development, involve reading texts of various genres, including narrative texts and poetry.

Texts of different genres vary in terms of their structural and lexical complexity. Research shows that text genre influences readers’ processing [10,11]. For instance, eye tracking studies demonstrated that the processing of narrative texts, as opposed to expository texts, is more challenging for young readers [12]. Unlike narrative texts, which typically present a sequence of events unfolding over time, expository texts often contain descriptions of concepts which might be verbalized by abstract or simply unfamiliar words. Moreover, gaps in prior knowledge and the knowledge of structural conventions [13,14] and deficits in inferential skills [15] might also hinder comprehension of expository texts. However, the identification of distinct strategies chosen by high-comprehending and low-comprehending readers when reading expository and narrative texts remains an open question. For instance, although expository texts are generally more difficult to process, the difference in processing of expository texts between low- and high-comprehending readers was found to be attenuated as compared to narrative texts which induced prolonged processing for low-comprehending readers [1].

Research demonstrated that prose and poetry are processed differently as well [16] due to specific characteristics of poetry including rhyme, rhythm, and intentional line breaks. It was found that poetry processing, unlike prose processing, is associated with slower reading, more regressive eye movements as well as longer fixations [17]. However, at present, studies conducted on samples of adults or students prevail [16,17,18]. Thus, there is a lack of research which concerns the processing of poetry as compared to other genres by children and adolescents.

### 1.2. Scanpath Analysis in the Field of Reading

A vast body of research in the field of reading has employed eye tracking methods as the main instrument [19,20,21]. Being an objective and non-invasive tool, eye tracking has become an exceptionally valuable technology for assessing visual perception. A significant amount of knowledge in the field of eye tracking studies focused on reading processes has been accumulated due to the research on language-related challenges. For instance, eye tracking technology is widely used as a tool for diagnosing dyslexia in children [22]. Eye movement metrics, i.e., fixation durations, fixation counts, and saccadic patterns, are routinely utilized to identify anomalies that distinguish populations facing language-related challenges from typical reading behaviors, thereby aiding in early screening. A recent study which was focused on foreign language vocabulary recognition in children with Attention Deficit/Hyperactivity Disorder (ADHD) and Autism Spectrum Disorder (ASD) has found that eye tracking technology can reveal specific attentional and processing patterns in neurodivergent children during language learning [23]. For instance, this study demonstrated that children with ADHD exhibited increased fixations on phonological distractors, while children with ASD showed more distributed attention. Research on language-related difficulties has shown that eye tracking can provide valuable insights into neurodiversity and language acquisition. However, it can also be used to study reading processes in average readers with differing levels of reading efficiency [24]. Moreover, the impact of various linguistic and individual factors on the reading process can also be studied through the eye movements [25].

Oculomotor performance can be analyzed at local and global levels. At the local level, each word is assigned to a specific area of interest, and changes in oculomotor parameters are evaluated at the word level. This approach provides rich information about the single-word level processing, but it has limitations when making the interpretation beyond this area of interest. The global level involves assessing changes in the whole eye movement pattern when reading sentences or text. Both of these levels can legitimately tell us about the process of perception, but when working with text, it is preferable to have a broader perspective and view of the problem. Thus, when the objective of research is to investigate the time-course changes in overall reading patterns, scanpaths appear to be one of the primary sources of data. Scanpath is a sequence of fixations represented by the location (x and y coordinates) and the duration of each fixation. This parameter reflects changes in the entire reading pattern, including regressions to specific areas of the text and the frequency of these regressions. This, in turn, provides the understanding of how oculomotor patterns differ in accordance with individual language proficiency [26], reading ability level [27], task specificity [3], text or sentence structure [28,29], disabilities [30]. Hence, using the global level analysis approach is necessary for catching the changes in eye movement reading patterns during the reading of texts of different genres, as well as understanding the impact of individual differences in reading comprehension on the reading process. It is important to note that eye tracking technology can be seen as a medium that supports the study of cognitive processes during reading, including reading comprehension. However, eye movements are a measure of reading behavior and only reflect cognitive processes, particularly reading comprehension [31,32].

To extend the research corpus of studies on how textual aspects, specifically text genre, influence reading patterns, we aimed to analyze scanpaths during the reading of texts of different genres. Additionally, we explored how individual differences in text comprehension moderate these reading patterns. It is worth noting that the current study was a continuation of our previous work [27], which directly aimed to address the previously identified limitations of the experimental design by implementing more natural reading conditions involving stimulus material and task type.

Our study addressed two main research questions:

1. Do reading patterns vary by text genre? Based on the previous studies, we hypothesized that expository texts would elicit a different scanpath as compared to narrative texts. Specifically, we expected that reading of the narrative text would be characterized by a more fluent reading pattern with fewer regressions and a faster reading process. We also expected poetry to generate distinct reading patterns relative to both expository and narrative texts due to its unique structure and stylistic features.
2. How do individual differences in text comprehension influence reading patterns? We hypothesized that a higher reading comprehension level would increase the likelihood of employing more fast and fluent reading patterns with fewer regressions across all genres.

## 2. Materials and Methods

### 2.1. Participants

The sample of the study included 44 adolescents aged 12 to 17 years (Mean age = 14.68, SD = 1.51, 23 female). All participants had normal or corrected vision and reported no history of mental disorders, language impairment, drug abuse or neurological diseases. Two participants reported experiencing hearing impairment. All participants were recruited locally through schools, social media posts, and distributed flyers. Details on participants’ demographics, reading habits, and language environment are provided in Appendix A Table A1 and Table A2.

### 2.2. Stimuli

The stimuli consisted of expository (*n* = 1; 166 word forms), narrative (*n* = 1; 166 word forms), and poetic (*n* = 1; 63 word forms) texts.

Narrative and expository texts in the abridged version were taken from the open bank of tasks for assessing the reading literacy of middle and high school students: https://oge.fipi.ru/bank/index.php?proj=B37230251B44AD1E4D5A616C96945D28 (accessed on 31 August 2025); the poetic text, according to the official program, is read in grade 10, but adolescents often read it earlier as part of their preparation for the state exam taken in grade 9. Taking into account that native speaker schoolchildren read texts of different complexity, we decided to retain the naturalistic situation rather than match the texts’ complexity by using complexity indices. Such parameters as interest or prior knowledge of the topic were not taken into account, but the fact that the selected texts were taken from the open task bank for schoolchildren as well as from the school program suggests that an average student should be familiar with the topics of the stimulus texts.

Moreover, in a naturalistic situation, texts could rarely be unambiguously attributed to the narrative or expository genre. Rather, there are texts where elements of the narrative or expository genre predominate. Therefore, in this case, by a narrative text we mean a text where narrative elements predominate, and by an expository text we mean a text where expository elements predominate. Nevertheless, narrative and expository elements differ significantly in structure. Texts where narrative elements predominate describe a sequence of events related to each other both temporally and causally. Texts with expository elements provide factual information concerning a certain concept. Expository text elements are more descriptive than narrative text elements. Poems use rhythmic and figurative language to convey certain ideas of the author.

For each of these three texts, five closed-ended and one open-ended question were developed. Closed-ended questions corresponded to cognitive processes described in PISA 2018 reading framework [33]. The first question for all texts was aimed at assessing the ability to comprehend literal information which is explicitly given in the text. Two subsequent questions were assessing the ability to make bridging inferences and knowledge-based inferences. In order to answer these types of questions, several operations were required from the reader. Bridging inferencing required linking parts of the text and using knowledge of linguistic operators. Knowledge-based inferences are critical to get an overall meaning of the text. Thus, it required the reader to be familiar with relevant vocabulary representing key concepts described in the text including the knowledge of plausible synonymous substitutions. The fourth and the fifth questions assessed the ability to evaluate and embed information which is provided in the text in a broad context: elaborative inferencing and evaluative inferencing. Elaborative inferencing required interpretation in terms of the reader’s existing knowledge, as well as making predictions by synthesizing information from the text and life experience. Evaluative inferencing involved emotional evaluation of the information provided in the text. For each correct answer to a closed-ended question, the participant received one point.

The open-ended question was similar for all three texts. It required the participants to describe in four sentences the essence of the given text. To check the answers to open-ended questions, assessment criteria were developed. Stimuli texts, tasks and assessment criteria for open-ended questions are available in the Open Science Framework (OSF) storage of the project: https://osf.io/9p3xh/ (accessed on 31 August 2025).

### 2.3. Procedure

The experimental session started with a brief introduction, during which the participants were informed about the tasks and procedures. Prior to the main part of the experiment, participants completed a training task which was not included in the subsequent analysis. Before the reading of each text, the equipment was calibrated and validated to ensure eye-tracking registration accuracy—participants were instructed to follow a moving point on the computer screen. The experimental procedure with eye tracking took approximately 30 min in total.

**Figure 1:**
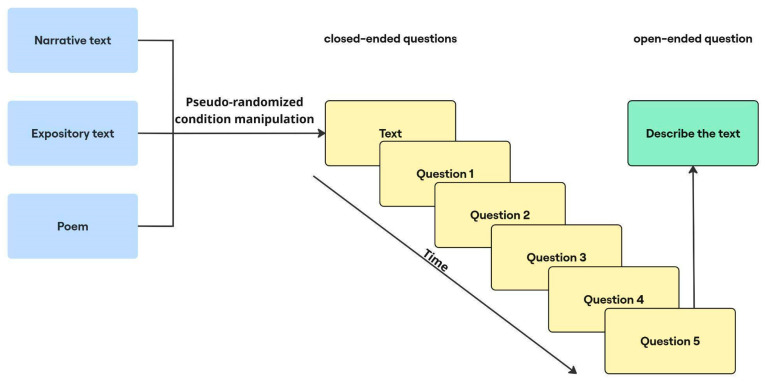
The scheme of the experimental design.

Participants completed three cycles, each consisting of a block of closed-ended questions immediately followed by an open-ended question (see Figure 1). First, the participants read a text associated with one of the three genres—narrative, expository or poetic—in pseudo-randomized order, and answered related closed-ended questions, with the option of referring back to the text. Afterwards, they summarized the main idea of the same text in four sentences (an open-ended question). This sequence ensured that every genre appeared once across the three repetitions. Before starting the main task, participants were asked to complete a training session in which they could familiarize themselves with all the stages. This training session was not included in the subsequent analysis.

### 2.4. Apparatus and Recordings

The experiment was conducted in a sound-attenuated room, with participants seated about 75 cm from the display monitor. To reduce head movement, a chin and forehead rest was used. Stimuli were displayed in black, 22-point Arial font against a light gray background using Experiment Builder software (Version 2.5.90; SR Research Ltd., Ottawa, ON, Canada). A Lenovo Legion monitor (144 Hz, 1920 × 1080 resolution) connected to a computer was used for presentation. Eye movements were tracked at 1000 Hz using the EyeLink 1000+ system (SR Research, Toronto, ON, Canada), recording the right eye only.

### 2.5. Data Pre-Processing

The eye-movement data were pre-processed in Data View (SR Research, Toronto, ON, Canada). Participants had the opportunity to return to the text while answering the first five questions. However, since our research questions focused on reading strategies employed during the initial encounter with the text, we analyzed scanpaths only for the period of first reading before moving on to the questions. During pre-processing, the data underwent visual inspection to ensure data quality and accurate mapping of fixations to the Areas of Interest (AOIs). Participants and items with poor calibration quality of eye-tracking data were excluded. As a result we excluded the 3.03% of trials. Furthermore, fixations that were slightly misaligned with their AOIs due to minor calibration drift were manually corrected. All fixations shorter than 60 ms were excluded from the analysis [34]. Each word of the text was marked as the independent area of interest.

### 2.6. Data Analysis

The obtained data were analyzed using the R programming language in R Studio version 1.1.2024 [35].

To analyze the influence of text genre on reading comprehension and the number of returns to the text, as well as the relationship between reading comprehension, the number of returns to the text, and the participant’s age, we used linear mixed-effects models implemented in the lme4 package [36]. Two models were run in total. In the first model, the dependent variable was the reading comprehension score, which was calculated by adding together the number of correct responses to both closed and open-ended questions. Text genre, number of returns to the text and age were entered as fixed effects. In the second model, the dependent variable was the number of returns to the text. Text genre, reading comprehension score and age were entered as fixed effects.

The analysis of oculomotor characteristics was divided into several key steps. First, it was necessary to calculate scanpaths and identify clusters. This stage was essential for determining the global reading pattern employed by the participants when reading texts of different genres. Next, linear regression was used to analyze differences between clusters to describe them. In the final stage, logistic regression was applied to examine the relationship between reading patterns and genre, while also considering the roles of text comprehension level and age. The data and analysis code for this study can be obtained by contacting the corresponding author. The original studies or the analysis plan of the present study were not preregistered.

For the first step, we employed a pipeline previously introduced in studies investigating reading patterns with scanpath analysis [26,27,37,38]. Using the scanpath package [39], we calculated pairwise dissimilarity scores between all scanpaths for each text. Due to differences in text length, a normalization procedure was applied to calculate the dissimilarity scores, which reduced the penalty scores for scanpaths with different lengths. Next, to assess the similarity or difference between participants’ scanpaths, we calculated scanpath maps using multi-dimensional scaling (MDS) with the MASS package [40]. We transformed the dissimilarity scores into several-dimensional representations of scanpath space. In these maps, spatial location encodes similarity: scanpaths with similar patterns appear next to each other, while divergent scanpaths appear further apart. We set the number of dimensions to 6, balancing stress values (4.9%) with preventing overfit [38]. Next, using the maps generated in the previous step, we identified clusters of similar reading patterns employing the mcclust package [41]. The optimal number of clusters was determined using an initial procedure of clustering without controlling the number of clusters selected. The median number for all texts was two clusters, which was the final decision for clustering.

Next, we analyzed differences in oculomotor characteristics between the identified clusters. This analysis was conducted using linear mixed-effects models implemented in the lme4 package [36]. The cluster was included as a fixed effect with dummy coding contrast, where the intercept represents the values for cluster 1. The participants and texts served as random factors. Since the research questions were related to the investigation of the whole reading pattern, we, following Blohm et al. [16], chose variables related to the global level as dependent variables: average amplitude of right saccades, average amplitude of left saccades, percentage of regressive saccades, rating rate, fixation rate. Separate models were fitted for each dependent variable. Such eye movement parameters were robust to the length of the text, which gave us the opportunity to compare texts of different genres.

In the final stage of analysis, we examined the relationships between cluster membership, text genre, comprehension scores and age. We constructed two models with cluster membership as the dependent variable, using the main effect and interaction between text genre, text comprehension score and age as the fixed factors.

In all models, continuous variables such as the reading comprehension score, the number of returns to the text, and the age of participants were centered and scaled. Texts’ genres were coded using sum-to-zero contrasts via the contr.sum() function in R [42], meaning that the intercept represented the grand mean across texts’ genres. All models included participants as random factors to account for individual differences. The resulting estimates represented fixed effects (β) in the models [36]. Pairwise contrasts were adjusted using the Bonferroni correction.

## 3. Results

### 3.1. Reading Comprehension, Number of Returns to Texts, Participant’s Age and Text Genre

**Figure 2:**
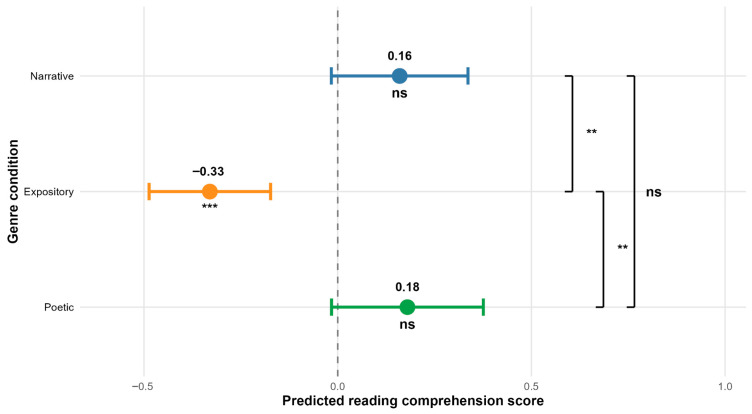
Effects of text genre on reading comprehension scores. Points represent model estimates (deviations from the intercept), and error bars show 95% confidence intervals. Asterisks indicate statistical significance: *** *p* < 0.001, ** *p* < 0.01, ns = not significant. Brackets on the right indicate pairwise comparisons between genres with Bonferroni correction. The dashed line represents the model intercept (grand mean across text genres when using sum-to-zero contrasts).

**Figure 3:**
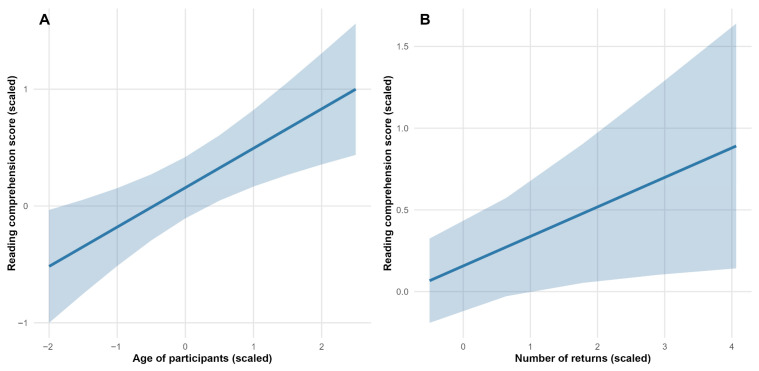
Association between (**A**) reading comprehension score and age of participants, and (**B**) reading comprehension and number of returns. All variables are scaled. Solid lines represent the predicted relationship from the linear mixed model; shaded areas indicate 95% confidence intervals.

The findings of this study indicated a statistically significant decline in the accuracy while completing text comprehension tasks corresponding to the expository text (β = −0.33, SE = 0.08, t-value = −3.95) (see Figure 2). We also found that participants’ reading comprehension increased with age (β = 0.34, SE = 0.10, t-value = 3.33) (see Figure 3A). We found increases in reading comprehension with an increasing number of returns to the text (β = 0.18, SE = 0.08, t-value = 2.24) (see Figure 3B). All model outputs are presented in Table 1.

Pairwise comparisons showed that accuracy while completing tasks related to the narrative text was significantly higher as opposed to the tasks associated with the expository text (β = 0.49, SE = 0.14, t-ratio = 3.40) (see Table 2, Figure 2). However, performance in tasks related to the expository text was significantly lower than in tasks corresponding to the poetic text (β = −0.51, SE = 0.16, t-ratio = −3.18) (see Table 2, Figure 2). The analysis revealed no statistically significant differences in results associated with tasks corresponding to narrative and poetic texts (see Table 2, Figure 2).

**Figure 4:**
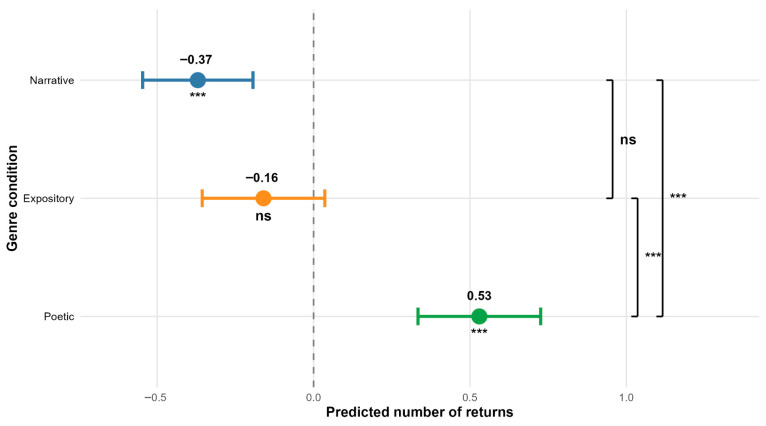
Effects of text genre on returns to text. Points represent model estimates (deviations from the intercept), and error bars show 95% confidence intervals. Asterisks indicate statistical significance: *** *p* < 0.001, ns = not significant. Brackets on the right indicate pairwise comparisons between genres with Bonferroni correction. The dashed line represents the model intercept (grand mean across text genres when using sum-to-zero contrasts).

**Figure 5:**
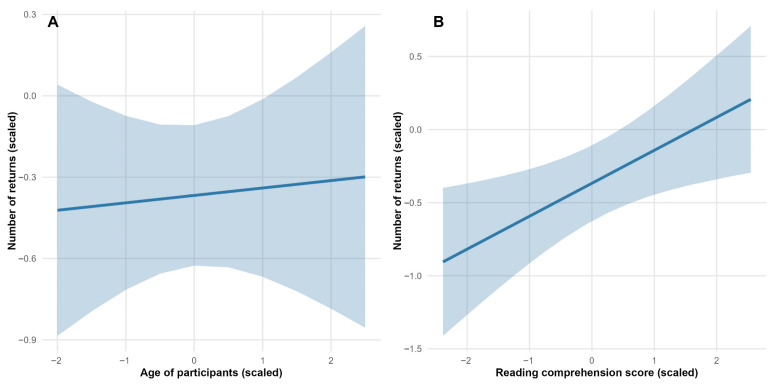
Association between number of returns and (**A**) age and (**B**) reading comprehension score. All variables are scaled. Solid lines show predicted relationships from the linear mixed model; shaded areas represent 95% confidence intervals.

Participants made significantly fewer returns to the narrative text (β = 0.00, SE = 0.09, t-value = −3.98) and more returns to the poetic text (β = 0.53, SE = 0.10, t-value = 5.51) (see Figure 4). The analysis revealed no significant effect of participants’ age on the number of returns to texts (see Table 3, Figure 5A). More returns to texts were associated with greater reading comprehension (β = 0.23, SE = 0.09, t-value = 2.54) (see Figure 5B).

Pairwise comparisons showed that there were significantly fewer returns in the case of the narrative text as opposed to the poetic text (β = −0.89, SE = 0.16, t-ratio = −5.50) and in the case of the expository text as opposed to the poetic text (β = −0.68, SE = 0.17, t-ratio = −3.96), with no difference between narrative and expository texts (see Table 4, Figure 4).

### 3.2. Eye Movement Analysis

#### 3.2.1. Scanpath Clusters

**Figure 6:**
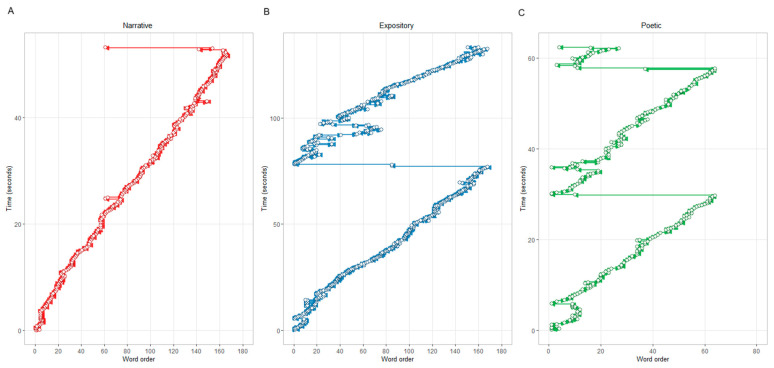
Visualization example of individual reading patterns across three genres (Participant BG3210). Panels show scanpaths for: (**A**) narrative, (**B**) expository, and (**C**) poetic texts. In all graphs, the *x*-axis represents word order and the *y*-axis represents the time course of the reading process. White circles represent fixations, vertical scanpath lines represent fixation duration, and horizontal lines represent forward and regressive saccades.

**Figure A1:**
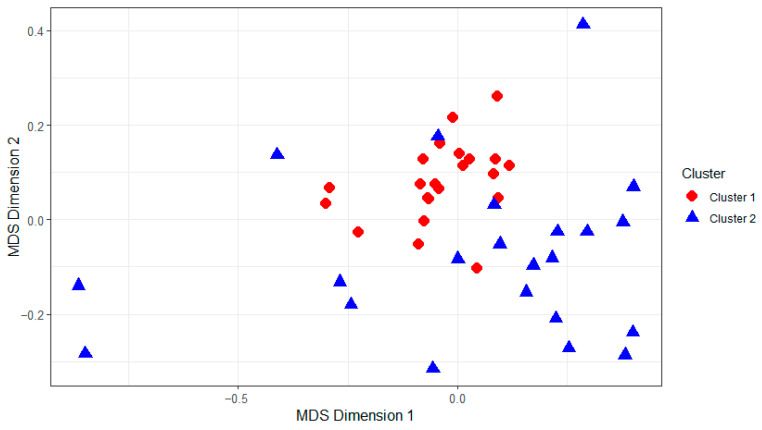
Scanpath clusters (projection onto the first two MDS axes). Points represent individual scanpaths; colors indicate cluster membership. Cluster 1 (red) = 47.66% of cases; Cluster 2 (blue) = 52.34% of cases. Proximity on the plot reflects scanpath dissimilarity (closer = more similar).

**Figure A2:**
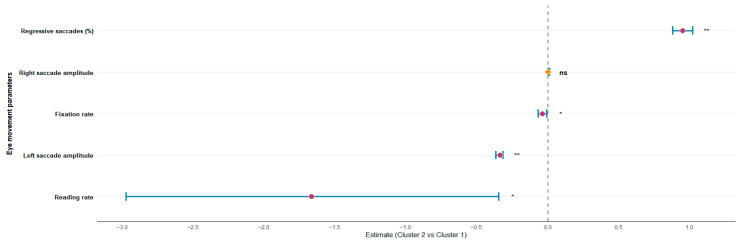
Forest plot of fixed effects for Cluster 2 membership on eye movement parameters. Estimates represent differences between Cluster 2 and Cluster 1 (reference group). Error bars indicate 95% confidence intervals. Asterisks indicate statistical significance: ** *p* < 0.01, * *p* < 0.05, ns = not significant. The dashed line represents the model intercept (cluster 1).

In the first stage, two clusters with distinct reading patterns were identified (the clustering procedure is detailed in the “Data Analysis” Section). The cluster diagrams are shown in Figure A1. Mean values for eye-tracking metrics are presented in Table 5. The first pattern included 47.66% of cases, while the second reading pattern included 52.34% of cases. Statistical comparisons with linear mixed-effects models revealed that Cluster 1 exhibited a significantly lower percentage of regressions (β = 0.95, SE = 0.03, t-value = 27.59) with larger regression amplitudes (β = −0.34, SE = 0.01, t-value = −27.30). Furthermore, Cluster 1 showed significantly higher values for reading rate (β = −1.67, SE = 0.67, t-value = −2.49) and fixation rate (β = −0.04, SE = 0.01, t-value = −2.64), suggesting slower text processing. In contrast, Cluster 2 demonstrated a higher percentage of shorter regressions and faster text processing compared to Cluster 1. Thus, Cluster 1 could be labeled as a forward reading pattern, while Cluster 2 represents a regressive reading pattern. Visualization of typical scanpaths is represented in Figure 6. Results of linear mixed-effect models of cluster comparison are provided in Table 6 and visualized in Figure A2.

#### 3.2.2. Association Between Text Genre and Text Comprehension

**Figure 7:**
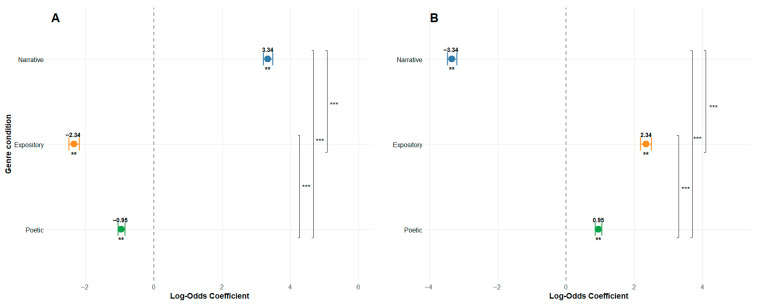
Effects of text genre on reading pattern clusters. The figure presents point estimates (log-odds coefficients) and 95% confidence intervals for the effects of three genre conditions (Narrative, Expository, Poetic) on two distinct reading pattern clusters: (**A**) Forward Reading Pattern and (**B**) Regressive Reading Pattern. Asterisks indicate statistical significance: *** *p* < 0.001, ** *p* < 0.01, ns = not significant. Brackets on the right indicate pairwise comparisons between genres with Bonferroni correction. The dashed line represents the model intercept (grand mean across text genres when using sum-to-zero contrasts).

**Figure 8:**
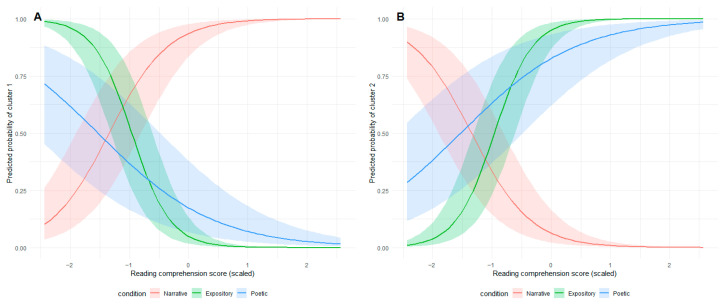
Predicted probabilities of reading pattern cluster membership as a function of reading comprehension ability and genre condition. Panel (**A**) shows the probability of Cluster 1 (forward reading pattern) membership, while Panel (**B**) shows the probability of Cluster 2 (regressive reading pattern) membership. Shaded areas represent 95% confidence intervals. The plots illustrate how the relationship between reading comprehension and cluster membership probability varies across different genre conditions (Narrative, Expository, Poetic).

To analyze relationships between clusters and text genre, reading comprehension, and age, we employed mixed-effects logistic regression Models. The analysis revealed a significant main effect of text genre on assignment to Cluster 1. Specifically, the probability of using the forward reading pattern (Cluster 1) increased for narrative texts (b = 3.34, SE = 0.07, z-value = 49.52) but decreased for expository (β = −2.34, SE = 0.08, z-value = −29.99) and poetic texts (β = −0.95, SE = 0.05, z-value = −18.76) (see Table 7, Figure 7A). A significant negative main effect of comprehension scores was also observed (β = −0.44, SE = 0.08, z-value = −5.71), indicating that higher comprehension scores were associated with reduced probability of using this pattern. Notably, we found a significant interaction between genre and comprehension. For narrative texts, higher comprehension scores increased the probability of using a forward reading pattern (β = 2.81, SE = 0.07, z-value = 41.31) (see Table 7, Figure 8A). For expository and poetic texts, higher comprehension scores reduced the probability of using a forward reading pattern (see Table 7, Figure 8A). Results of the mixed-effects logistic regressions are presented in Table 7 and visualized in Figure 8. The results of pairwise comparisons are presented in Table 8.

Conversely, for expository texts, the interaction decreased the probability of using this pattern (β = −2.19, SE = 0.07, z-value = −29.83). Conversely, inverse effects emerged for Cluster 2 (the regressive reading pattern). The probability of using this pattern significantly decreased for narrative texts (β = −3.34, SE = 0.07, z-value = −49.53) but increased for expository (β = 2.34, SE = 0.08, z-value = 29.99) and poetic texts (β = 0.95, SE = 0.05, z-value = 18.76) (see Table 7, Figure 7B). Higher reading comprehension scores significantly predicted a greater likelihood of using the pattern of Cluster 2 (β = 0.44, SE = 0.08, z-value = 5.71). Critically, the interaction between text genre and level of comprehension was also significant. For narrative texts, higher comprehension reduced the probability of using regressive reading pattern (β = −2.81, SE = 0.07, z-value = −41.31) (see Figure 7B), whereas for expository texts, higher comprehension increased this probability (β = 2.19, SE = 0.07, z-value = 29.83) (see Figure 8B), and for poetic texts, higher comprehension also increased this probability (β = 0.32, SE = 0.05, z-value = 6.37) (see Figure 8B). Age did not significantly predict cluster membership in any models. Results of the mixed-effects logistic regressions are presented in Table 7 and visualized in Figure 8. The results of pairwise comparisons are presented in Table 8.

## 4. Discussion

### 4.1. Reading Comprehension, Number of Returns to Text, Participant’s Age and Text Genre

In our study, we showed that narrative text is easier to comprehend than expository text, thereby corroborating the findings of several earlier investigations synthesized in the meta-analysis by Clinton et al. [43]. This difference likely arises because narrative texts are organized around characters’ goals and a chronological sequence, which helps readers anticipate logical connections among ideas, whereas expository texts employ a more varied structure that hinders their processing; furthermore, the vocabulary of narrative texts is organized more simply—both in terms of word length and usage frequency—than that of expository texts.

We found that a higher number of returns to the text for rereading predicted better reading comprehension. This result aligns with previous studies demonstrating that increased rereading reflects deeper text processing and leads to improved comprehension. Notably, blocking the opportunity to reread previously read text has been shown to negatively affect comprehension [44]. Similarly, Strukelj and Niehorster [45] observed that thorough reading—characterized by longer total reading times and more rereading—resulted in higher comprehension scores compared to regular reading, skimming, or spell-checking tasks. These findings underscore the importance of revisiting textual content for constructing coherent meaning representations. In addition, we found that there was a greater number of returns to the text in the poetry text condition, which on one hand may indicate a need for deeper processing of the abstractions inherent in poetry. Interpreting poetry often requires additional cognitive effort on the part of the reader, as poetry is often characterized by figurative language. In other words, poetry cannot always be understood if interpreted literally. Thus, rereading may indicate additional cognitive operations that the reader performs in order to understand metaphors and other stylistic devices. However, figurative language often allows poetry to be interpreted in different ways. Understanding of poetry depends on individual differences and according to their life experiences and preferences [17]. In narrative and expository texts, the number of interpretations is limited, as the linguistic means used in these texts do not allow for such variability in terms of possible interpretations. For the narrative text, the number of returns to the text decreased, which may indicate a simpler structure of the narrative text that does not require deeper processing.

Our results showed a positive correlation between age and reading comprehension in the adolescent group, suggesting that as age progresses, reading comprehension levels also increase. This finding is consistent with the findings of numerous studies that have demonstrated that older adolescents employ reading strategies that facilitate enhanced comprehension [46,47]. In our previous work, Berlin Khenis et al. [27] also demonstrated a positive correlation between the age of participants within the adolescent group and their working memory and vocabulary scores. This may also explain why reading comprehension increased with age in the current study.

### 4.2. Eye Movement Patterns

During the eye movement analysis, we identified two groups of scanpaths. Based on the combination of eye movement characteristics, we classified the reading patterns in the first group as the forward reading patterns and those in the second group as regressive reading patterns. This classification arose from the observation that the first group is represented by the reading patterns with fewer regressions with greater amplitude, along with higher reading and fixation rates. In contrast, the second group is characterized by patterns with a higher number of regressions with shorter amplitude and lower reading and fixation rates. Analyzing the relationship between these clusters and text genres, the level of reading comprehension and age, we found that the likelihood of employing forward reading patterns increases when reading narrative texts. Moreover, a higher comprehension score of such texts enhances this tendency. Interestingly, expository and poetic texts have a negative effect on the probability of using this pattern, and higher comprehension scores only increased this effect. Conversely, the use of the regressive reading pattern becomes more likely when reading expository and poetic texts and this tendency also grows with enhancing comprehension of these texts. In contrast, narrative texts exhibit the opposite effect, reducing the probability of using such patterns. Thus, following our first research question we confirm that reading patterns vary depending on the text genre. However, the answer to the second research question was less clear and contradicted initial expectations. Contrary to the hypothesis that increased comprehension would lead to an overall reduction in regressions, we found that comprehension modulated different reading patterns (forward or regressive reading pattern) depending on genre. Moreover, the overall trend, independent of genre, showed that increased reading comprehension generally increased the likelihood of the regressive reading pattern and decreased the likelihood of using forward reading patterns.

Many models of discourse comprehension focus on examining the nature of mental representations that emerge during reading [48]. Text comprehension depends on multiple factors, including genre and associated with genre expectations, which are based on previously acquired knowledge about textual structures, genre-specific reading strategies, genre functions, and communicative purposes [49,50,51,52]. Genre expectations may influence the reading strategies which readers employ when approaching a text. Furthermore, as previous research has demonstrated, such strategic choices may be determined by accumulated experience with a particular genre, its typical text length or genre-specific coherence expectations, all of which affect which reading strategies are adopted [52,53,54]. Thus, the patterns we identified—distinguished by oculomotor activity and showing differential probabilities of being employed with different text genres—fit well within this theoretical framework.

Our results indicate that expository and poetic texts increase the likelihood of employing the regressive reading pattern, which is characterized by a higher frequency of regressions compared to forward reading patterns. Current research associates regressive eye movements with problem-solving strategies during reading, whether related to lexical access difficulties, syntactic processing challenges, or discourse-level comprehension issues [44,55,56,57,58]. Furthermore, recent scanpath analysis studies have demonstrated that reading patterns with more regressions tend to correlate with a lower level of text comprehension skill [3,27]. This suggests that the adoption of regressive reading patterns may reflect attempts to overcome comprehension difficulties. However, previous studies have shown that there are differences in reading strategies between prose and poetry. Specifically, several studies have found a difference in reading time, with poetic texts being read more slowly, and in eye movement patterns, with more regressions when reading poetry as compared to prose [16,17]. Given these established differences between poetic and prose genres, we hypothesize that the underlying cognitive mechanisms driving these regressions likely differ between genres.

As previously discussed, studies have identified consistent differences in processing strategies, as well as in the speed and accuracy of comprehension, between expository and narrative genres. Schmitz et al. [59] suggest that the differences they observed in text comprehension between these genres may largely be due to variations in global cohesion. The authors build on the claim that reading literary fiction is typically associated with pleasure [53] and operates in a “wait-and-see” mode [51]. Readers naturally construct causal connections as they progress through the text [60], integrating fragments into a unified mental representation. Despite the narrative genre used in our study not being directly related to literature, the coherence effects highlighted by Schmitz et al. [59] may still explain the likelihood of distinct reading patterns for these genres. Thus, the higher frequency of regressive eye movements in expository texts might reflect not merely their greater complexity compared to narratives, but specifically their lower coherence, which requires readers to revisit earlier segments to establish connections independently.

The influence of text coherence can also be applied to poetic texts as well, since processing metaphorical language and figurative devices may challenge the construction of propositional text models. However, when examining poetry comprehension, we must account for the additional effects of meter and rhythm [18,61,62] as well as layout used in the traditional presentation of poetic texts [63,64]. Early theoretical work proposed that text comprehension can be explained through the structural building framework [65,66]. This framework posits that reading comprehension involves constructing a mental structure of the text. During initial reading stages, readers establish a foundation for these mental structures, which they subsequently develop by creating coherent information maps that connect new input with prior context. Blohm et al. [16] suggested that during the early stages of poetry reading, readers form a rhythmic pattern foundation, which facilitates better rhythm perception as they progress through the poem. In contrast, when reading prose, after establishing an initial propositional model, readers may adopt a riskier strategy, where readers skip more words and progress faster through the text. This occurs because the established discourse model provides thematic constraints that enable faster processing as readers encounter more contextual information. This logic may also apply to silent reading, potentially explaining the differences between the reading patterns we identified. However, the exact mechanisms behind regressive patterns in poetry reading remain unclear. Beyond the challenges posed by metaphorical language processing, we must consider rhythmic and metrical influences. Beck and Konieczny [62] demonstrated that metrical anomalies increased rereading, while Menninghaus and Wallot [18] found no global changes in eye movement patterns when varying rhythm and meter conditions. Interestingly, they observed fewer regressions when readers gave high esthetic ratings, suggesting that esthetic appreciation may influence poetry reading strategies. Additionally, layout may contribute to poetry’s higher regression frequency compared to other genres. Fechino et al. [64] compared eye movement characteristics when reading sonnets presented in either poetic or prose layout. The original poetic layout led to an increase in the number of regressions. The authors suggested that the initial categorization of the text, based on the reader’s experience and expectations of the structure of the text, influences their reading strategy.

Previous research has identified that a faster, more linear reading pattern with fewer regressions is typically associated with reading expository texts [57,67]. However, as discussed earlier, increased regression frequency generally indicates the use of reading strategies when encountering text difficulties. The finding that both expository and poetic texts show increased probability of regressive reading patterns when comprehension is considered provides further support for this interpretation. Drawing on different models of discourse comprehension (for instance, the Construction–Integration Model, the Structure-Building Framework, or the Landscape Model [48]), our findings reflect an active reading process in which readers strive to construct a coherent mental representation of the text. In the case of narrative texts, which, as noted earlier, are typically organized around characters’ goals and a chronological sequence that helps readers anticipate logical connections among ideas, readers appear to build this representation relatively automatically, as evidenced by a forward reading pattern. In contrast, for expository and poetic texts—due to the specific challenges outlined above—readers experience greater difficulty in constructing a coherent representation. Under these conditions, they resort to an increased number of regressions, which facilitates the integration of textual elements into a unified mental model and, consequently, enhances comprehension. Notably, the comprehension factor itself demonstrated a significant main effect, increasing the likelihood of using a regressive reading pattern, which is consistent with prior research. This suggests that while genre characteristics and genre-based expectations undoubtedly influence reading strategy selection, the actual level of text comprehension achieved also plays a crucial role in modulating reading patterns.

The identified clusters differed in reading rate and fixation rate, indicating that texts read with the forward reading pattern were processed longer than those read with the regressive pattern. Previous research has shown that more regressions typically indicate a pattern where readers resolve comprehension difficulties, which is usually associated with increased reading time. Our results contradict these earlier findings. However, although the difference between clusters in reading rate and fixation rate reached statistical significance, the effect size was extremely small. Therefore, its interpretation remains questionable and requires further verification.

### 4.3. Limitations

The present study design has certain limitations and assumptions that should be considered when interpreting the results and planning future research. Previous studies have demonstrated that text topic, background knowledge, and personal preferences can significantly influence reading strategies and comprehension levels. We did not include these factors in the analysis. However, they represent a major issue that warrants dedicated investigation with consideration of genre-specific effects. Furthermore, the study was related to a single linguistic and cultural context and did not involve measures of cognitive factors like working memory or linguistic proficiency.

Although the stimulus materials were close to those that adolescents interact with at school, the use of the EyeLink equipment did not allow for the creation of a naturalistic setting. Reading with the chin and head fixed before a vertical screen might have influenced the results. Another limitation is that the study was conducted on a relatively small sample (N = 44).

The texts used in this study did not vary in terms of difficulty, though this factor is also important in reading analysis and genre research. We found that genres inherently differ in complexity regarding perception accuracy. However, it would be important to consider how reading patterns and text comprehension levels change depending on text difficulty within one genre.

Moreover, this study was focused on average adolescent readers. In future research, it would be worth analyzing whether the findings might extend to populations with reading difficulties or neurodiverse profiles.

## 5. Conclusions

In conclusion, our results demonstrate that reading texts of different genres involves distinct eye-movement patterns. However, although some genres exhibit similar oculomotor activity, the underlying reasons for increased regressive patterns may vary across genres. Furthermore, reading comprehension modulates the use of different eye-movement patterns in genre-specific ways, further supporting the existence of genre-dependent strategies. Thus, we conclude that text genre significantly influences the selection and adaptation of oculomotor strategies during reading. This factor should be accounted for in future studies investigating the relationship between eye-movement characteristics and reading comprehension.

## Figures and Tables

**Table 1:** Result of linear mixed-effects model on reading comprehension.

	Est (SE)	CI, 95%	t-Value	*p*-Value	Cohen’s d
Intercept	0.00 (0.10)	−0.20; 0.20	0.00	1.00	0.00
Narrative	0.16 (0.09)	−0.01; 0.33	1.82	0.07	0.27
Expository	−0.33 (0.08)	−0.50; −0.17	−3.95	**0.00**	−0.57
Poetic	0.18 (0.10)	−0.01; 0.36	1.86	0.07	0.30
Number of returns	0.18 (0.08)	0.02; 0.34	2.24	**0.03**	0.31
Age of participants	0.34 (0.10)	0.14; 0.54	3.33	**0.00**	0.57

Note: the results of logistic mixed-effect models, where the text genre, the number of returns to the text and age served as fixed effects. The text genres were coded as sum-to-zero contrast, and returns to the text and age were centered and scaled. Participants were used as random factors. Statistically significant *p*-values (*p* < 0.05) are marked in bold.

**Table 2:** Pairwise contrasts.

	Est (SE)	CI, 95%	t-Ratio	*p*-Value	Cohen’s d
Narrative-Expository	0.49 (0.14)	0.14; 0.84	3.40	**0.00**	0.74
Narrative-Poetic	−0.02 (0.16)	−0.42; 0.38	−0.11	1.00	−0.03
Expository-Poetic	−0.51 (0.16)	−0.90; −0.12	−3.18	**0.01**	−0.76

Note: pairwise contrasts adjusted using the Bonferroni correction. Statistically significant *p*-values (*p* < 0.05) are marked in bold.

**Table 3:** Result of linear mixed-effects model on returns to text.

	Est (SE)	CI, 95%	t-Value	*p*-Value	Cohen’s d
Intercept	−0.00 (0.09)	−0.18; 0.18	0.00	1.00	0.00
Narrative	−0.37 (0.09)	−0.55; −0.19	−3.98	**0.00**	−0.54
Expository	−0.16 (0.10)	−0.35; 0.03	−1.61	0.11	−0.23
Poetic	0.53 (0.10)	0.34; 0.71	5.51	**0.00**	0.70
Reading comprehension	0.23 (0.09)	0.05; 0.40	2.54	**0.01**	0.33
Age of participants	0.03 (0.10)	−0.17; 0.22	0.28	0.78	0.04

Note: the results of logistic mixed-effect models, where the text genre, reading comprehension and age served as fixed effects. The text genres were coded as sum-to-zero contrast, and reading comprehension and age were centered and scaled. Participants were used as random factors. Statistically significant *p*-values (*p* < 0.05) are marked in bold.

**Table 4:** Pairwise contrasts.

	Est (SE)	CI, 95%	t-Ratio	*p*-Value	Cohen’s d
Narrative-Expository	−0.21 (0.17)	−0.62; 0.20	−1.26	0.64	−0.28
Narrative-Poetic	−0.89 (0.16)	−1.29; −0.50	−5.50	**0.00**	−1.20
Expository-Poetic	−0.68 (0.17)	−1.10; −0.26	−3.96	**0.00**	−0.91

Note: pairwise contrasts adjusted using the Bonferroni correction. Statistically significant *p*-values (*p* < 0.05) are marked in bold.

**Table 5:** Descriptive statistics for eye movement parameters.

	Fast Reading Pattern, Mean (sd)	Non-Linear Slow Pattern, Mean (sd)
average amplitude of right saccades	3.24 (0.73)	3.22 (0.82)
average amplitude of left saccades	7.54 (1.73)	6.94 (2.46)
percentage of regressive saccades	28.87 (4.55)	30.54 (5.63)
reading rate	73.77 (34.5)	71.61 (32.33)
fixation rate	2.07 (0.81)	2.05 (0.8)

**Table 6:** Result of linear mixed-effects model on eye movement parameter.

	Est (SE)	t-Value	CI, 95%	*p*-Value	Cohen’s d
	Average amplitude of right saccades				
intercept	3.26 (0.26)	12.45	2.60; 3.95	0.001	
cluster2	0.00 (0.00)	0.99	−0.00; 0.01	0.32	−0.01
	Average amplitude of left saccades				
intercept	7.15 (1.02)	7.01	4.34; 9.96	0.005	
cluster2	−0.34 (0.01)	−27.30	−0.37; −0.32	**0.001**	0.40
	Percentage of regressive saccades				
intercept	30.11 (1.56)	19.33	26.06; 34.16	0.001	
cluster2	0.95 (0.03)	27.59	0.88; 1.02	**0.001**	−0.40
	Reading rate				
intercept	72.60 (2.24)	32.39	68.09; 77.10	0.001	
cluster2	−1.67 (0.67)	−2.49	−2.98; −0.35	**0.03**	0.05
	Fixation rate				
intercept	2.06 (0.05)	41.61	1.96; 2.16	0.001	
cluster2	−0.04 (0.01)	−2.64	−0.07; −0.01	**0.014**	0.05

Note: Results of linear mixed-effects model, where cluster was applied as a fixed factor with dummy coding. The intercept in all models represented the results of cluster 1. Participants and text were used as random factors. Statistically significant *p*-values (*p* < 0.05) are marked in bold.

**Table 7:** The results of the logical mixed-effect models.

	Est (SE)	CI, 95%	z-Value	*p*-Value	odd’s Ratio
Forward reading pattern (cluster 1)					
Intercept	−0.59 (0.57)	−1.67; 0.45	−1.03	0.30	0.55
Narrative	3.34 (0.07)	3.18; 3.43	49.52	**0.001**	28.32
Expository	−2.34 (0.08)	−2.49; −2.21	−29.99	**0.001**	0.09
Poetic	−0.95 (0.05)	−1.05; −0.85	−18.76	**0.001**	0.39
Reading comprehension	−0.44 (0.08)	−0.84; −0.56	−5.71	**0.001**	0.64
Age of participants	0.33 (0.55)	−0.58; 1.49	0.61	0.54	1.40
Narrative*reading comprehension	2.81 (0.07)	2.57; 2.83	41.31	**0.001**	16.67
Expository*reading comprehension	−2.19 (0.07)	−2.52; −2.24	−29.83	**0.001**	0.11
Poetic*text comprehension	−0.32 (0.05)	−0.42, −0.22	−6.37	**0.001**	0.73
Regressive reading pattern (cluster 2)					
Intercept	0.59 (0.57)	−0.44; 1.66	1.04	0.30	1.80
Narrative	−3.34 (0.07)	−3.43; −3.18	−49.53	**0.001**	0.03
Expository	2.34 (0.08)	2.21; 2.49	29.99	**0.001**	10.44
Poetic	0.95 (0.05)	0.85; 1.05	18.76	**0.001**	2.59
Reading comprehension	0.44 (0.08)	0.56; 0.84	5.71	**0.001**	1.55
Age of participants	−0.34 (0.55)	−1.48; 0.57	−0.61	0.54	0.71
Narrative*reading comprehension	−2.81 (0.07)	−2.83; −2.57	−41.31	**0.001**	0.06
Expository*reading comprehension	2.19 (0.07)	2.24; 2.52	29.84	**0.001**	8.93
Poetic*text comprehension	0.32 (0.05)	0.22; 0.42	6.37	**0.001**	1.38

Note: the results of logistic mixed-effect models, where the text genre, text comprehension and age served as fixed effects. The text genres were coded as sum-to-zero contrast, and text comprehension and age were centered and scaled. Participants and text were used as random factors. The asterisk (*) indicates an interaction effect between variables. Statistically significant *p*-values (*p* < 0.05) are marked in bold.

**Table 8:** Pairwise contrasts.

	Est (SE)	CI, 95%	z-Ratio	*p*-Value	odd’s Ratio
Forward reading pattern (cluster 1)					
Narrative-Expository	5.65 (0.13)	5.40; 5.90	44.90	**0.001**	284.43
Narrative-Poetic	4.25 (0.09)	4.07; 4.43	47.54	**0.001**	70.26
Expository-Poetic	−1.40 (0.11)	−1.61; −1.19	−13.04	**0.001**	0.25
Regressive reading pattern (cluster 2)					
Narrative-Expository	−5.65 (0.13)	−5.90, −5.40	−44.93	**0.001**	0.004
Narrative-Poetic	−4.25 (0.09)	−4.43, −4.07	−47.55	**0.001**	0.014
Expository-Poetic	1.40 (0.11)	1.19, 1.61	13.05	**0.001**	4.05

Note: pairwise contrasts adjusted using the Bonferroni correction. Statistically significant *p*-values (*p* < 0.05) are marked in bold.

## Data Availability

The data and analysis code for this study are openly available on the Open Science Framework: https://osf.io/9p3xh/ (accessed on 31 August 2025).

## Appendix A

**Table A1:** The participants’ demographic characteristics and reading habits.

Variable	*n*	%
Age		
12 years	7	15.90
13 years	12	27.30
14 years	5	11.40
15 years	11	25.00
16 years	7	15.90
17 years	2	4.50
Grade		
6th	8	18.18
7th	9	20.45
8th	7	15.91
9th	10	22.73
10th	6	13.64
11th	4	9.09
Gender		
Male	21	47.73
Female	23	52.27
Daily reading time (% of the typical day)		
<10%	4	9.09
from 10% to 20%	12	27.27
from 20% to 50%	19	43.18
>50%	9	20.45
Leading arm		
Left	5	11.36
Right	38	86.36
Both	1	2.27

**Table A2:** Language environment of the participants.

Language Environment	*n*	%
Infancy		
Only or mostly Russian	41	93.18
Equally Russian and another language	2	4.55
Mostly another language	0	0
Only another language	1	2.27
Preschool		
Only or mostly Russian	40	90.91
Equally Russian and another language	2	4.55
Mostly another language	2	4.55
Only another language	0	0
Elementary school		
Only or mostly Russian	35	79.55
Equally Russian and another language	9	20.45
Mostly another language	0	0
Only another language	0	0
Middle/High school		
Only or mostly Russian	32	72.73
Equally Russian and another language	12	27.27
Mostly another language	0	0
Only another language	0	0
Experience of living in non-Russian speaking environment		
yes	37	84.09
no	7	15.91

## References

[1] Kraal A., Koornneef A.W., Saab N., Van Den Broek P.W. (2018). Processing of Expository and Narrative Texts by Low- and High-Comprehending Children. Read. Writ..

[2] Rapp D.N., Broek P.V.D., McMaster K.L., Kendeou P., Espin C.A. (2007). Higher-Order Comprehension Processes in Struggling Readers: A Perspective for Research and Intervention. Sci. Stud. Read..

[3] Mézière D.C., Yu L., Reichle E.D., Von Der Malsburg T., McArthur G. (2023). Using Eye-Tracking Measures to Predict Reading Comprehension. Read. Res. Q..

[4] Van Dyke J.A. (2021). Introduction to the Special Issue: Mechanisms of Variation in Reading Comprehension: Processes and Products. Sci. Stud. Read..

[5] Perfetti C., Stafura J. (2014). Word Knowledge in a Theory of Reading Comprehension. Sci. Stud. Read..

[6] Duke N.K., Pearson P.D., Farstrup A.E., Samuels S.J. (2002). Effective Practices for Developing Reading Comprehension. What Research Has to Say About Reading Instruction.

[7] Schroeder S. (2011). What Readers Have and Do: Effects of Students’ Verbal Ability and Reading Time Components on Comprehension with and without Text Availability. J. Educ. Psychol..

[8] Kraal A., Van Den Broek P.W., Koornneef A.W., Ganushchak L.Y., Saab N. (2019). Differences in Text Processing by Low- and High-Comprehending Beginning Readers of Expository and Narrative Texts: Evidence from Eye Movements. Learn. Individ. Differ..

[9] Swales J.M. (1990). Genre Analysis: English in Academic and Research Settings.

[10] van den Broek P., Kendeou P. (2017). Development of Reading Comprehension: Change and Continuity in the Ability to Construct Coherent Representations. Theories of Reading Development.

[11] Oakhill J.V., Cain K. (2012). The Precursors of Reading Ability in Young Readers: Evidence From a Four-Year Longitudinal Study. Sci. Stud. Read..

[12] Best R.M., Floyd R.G., Mcnamara D.S. (2008). Differential Competencies Contributing to Children’s Comprehension of Narrative and Expository Texts. Read. Psychol..

[13] Oakhill J., Cain K., Elbro C. (2014). Understanding and Teaching Reading Comprehension: A Handbook.

[14] Meyer B.J.F., Ray M.N. (2011). Structure Strategy Interventions: Increasing Reading Comprehension of Expository Text. Int. Electron. J. Elem. Educ..

[15] Lorch R.F. (2017). Chapter 6—What Is so Difficult about Expository Text?. Reading Comprehension in Educational Settings.

[16] Blohm S., Versace S., Methner S., Wagner V., Schlesewsky M., Menninghaus W. (2022). Reading Poetry and Prose: Eye Movements and Acoustic Evidence. Discourse Process..

[17] Corcoran R., De Bezenac C., Davis P. (2023). ‘Looking before and after’: Can Simple Eye Tracking Patterns Distinguish Poetic from Prosaic Texts?. Front. Psychol..

[18] Menninghaus W., Wallot S. (2021). What the Eyes Reveal about (Reading) Poetry. Poetics.

[19] Chen Y., Guo Q., Qiao C., Wang J. (2025). A Systematic Review of the Application of Eye-Tracking Technology in Reading in Science Studies. Res. Sci. Technol. Educ..

[20] Wang T.N., Jian Y.C. (2022). A Systematic Review of Eye-Tracking Studies on Text-Diagram Science Reading. Bull. Educ. Psychol..

[21] Toki E.I. (2024). Using Eye-Tracking to Assess Dyslexia: A Systematic Review of Emerging Evidence. Educ. Sci..

[22] Andreou G., Argatzopoulou A. (2025). Investigating Foreign Language Vocabulary Recognition in Children with ADHD and Autism with the Use of Eye Tracking Technology. Brain Sci..

[23] Anderson N.C., Anderson F., Kingstone A., Bischof W.F. (2015). A Comparison of Scanpath Comparison Methods. Behav. Res..

[24] Spichtig A., Pascoe J., Ferrara J., Vorstius C. (2017). A Comparison of Eye Movement Measures across Reading Efficiency Quartile Groups in Elementary, Middle, and High School Students in the U.S. J. Eye Mov. Res..

[25] Clifton C., Staub A., Rayner K., Van Gompel R.P.G., Fischer M.H., Murray W.S., Hill R.L. (2007). Chapter 15—Eye Movements in Reading Words and Sentences. Eye Movements.

[26] Parshina O., Sekerina I.A., Lopukhina A., Von Der Malsburg T. (2022). Monolingual and Bilingual Reading Processes in Russian: An Exploratory Scanpath Analysis. Read. Res. Q..

[27] Berlin Khenis A., Markevich M., Streltsova A., Grigorenko E.L. (2024). Eye Movement Patterns in Russian-Speaking Adolescents with Differing Reading Comprehension Proficiency: Exploratory Scanpath Analysis. J. Intell..

[28] Von Der Malsburg T., Vasishth S. (2013). Scanpaths Reveal Syntactic Underspecification and Reanalysis Strategies. Lang. Cogn. Process..

[29] Olkoniemi H., Mézière D., Kaakinen J.K. (2024). Comprehending Irony in Text: Evidence from Scanpaths. Discourse Process..

[30] Ziubanova A.A., Laurinavichyute A.K., Parshina O. (2023). Does Early Exposure to Spoken and Sign Language Affect Reading Fluency in Deaf and Hard-of-Hearing Adult Signers?. Front. Psychol..

[31] Raney G.E., Campbell S.J., Bovee J.C. (2014). Using Eye Movements to Evaluate the Cognitive Processes Involved in Text Comprehension. J. Vis. Exp. JoVE.

[32] Andreou G., Gkantaki M. (2024). Tracking Adults’ Eye Movements to Study Text Comprehension: A Review Article. Languages.

[33] OECD (2019). PISA 2018 Reading Framework. PISA 2018 Assessment and Analytical Framework.

[34] Holmqvist K., Nyström M., Andersson R., Dewhurst R., Jarodzka H., van de Weijer J. (2011). Eye Tracking: A Comprehensive Guide to Methods and Measures.

[35] R: The R Project for Statistical Computing. https://www.r-project.org/.

[36] Bates D., Mächler M., Bolker B., Walker S. (2015). Fitting Linear Mixed-Effects Models Using Lme4. J. Stat. Softw..

[37] Mézière D.C., Yu L., McArthur G., Reichle E.D., Von Der Malsburg T. (2024). Scanpath Regularity as an Index of Reading Comprehension. Sci. Stud. Read..

[38] von der Malsburg T., Vasishth S. (2011). What Is the Scanpath Signature of Syntactic Reanalysis?. J. Mem. Lang..

[39] von der Malsburg T. *Scanpath: Tools for Analyzing Spatio-Temporal Patterns in Eye Movements [Computer Software]*, R Package Version 1.0.0; 2018. https://github.com/tmalsburg/scanpath/blob/master/scanpath/DESCRIPTION.

[40] Venables W.N., Ripley B.D. (2002). Modern Applied Statistics with S..

[41] Scrucca L., Fraley C., Murphy T.B., Raftery A.E. (2023). Model-Based Clustering, Classification, and Density Estimation Using Mclust in R..

[42] Schad D.J., Vasishth S., Hohenstein S., Kliegl R. (2020). How to Capitalize on a Priori Contrasts in Linear (Mixed) Models: A Tutorial. J. Mem. Lang..

[43] Clinton V., Taylor T., Bajpayee S., Davison M.L., Carlson S.E., Seipel B. (2020). Inferential Comprehension Differences between Narrative and Expository Texts: A Systematic Review and Meta-Analysis. Read. Writ..

[44] Schotter E.R., Tran R., Rayner K. (2014). Don’t Believe What You Read (Only Once): Comprehension Is Supported by Regressions During Reading. Psychol. Sci..

[45] Strukelj A., Niehorster D.C. (2018). One Page of Text: Eye Movements during Regular and Thorough Reading, Skimming, and Spell Checking. J. Eye Mov. Res..

[46] Kolić-Vehovec S., Bajšanski I. (2006). Metacognitive Strategies and Reading Comprehension in Elementary-School Students. Eur. J. Psychol. Educ..

[47] Denton C.A., Wolters C.A., York M.J., Swanson E., Kulesz P.A., Francis D.J. (2015). Adolescents’ Use of Reading Comprehension Strategies: Differences Related to Reading Proficiency, Grade Level, and Gender. Learn. Individ. Differ..

[48] McNamara D.S., Magliano J. (2009). Chapter 9 Toward a Comprehensive Model of Comprehension. Psychology of Learning and Motivation.

[49] Graesser A.C., McNamara D.S., Louwerse M.M., Sweet A.P., Snow C.E. (2003). What do Readers Need to Learn in Order to Process Coherence Relations in Narrative and Expository Text?. Rethinking Reading Comprehension.

[50] Schnotz W., Mayer R.E., Fiorella L. (2021). Integrated Model of Text and Picture Comprehension. The Cambridge Handbook of Multimedia Learning.

[51] van Dijk T.A., Kintsch W. (1983). Strategies of Discourse Comprehension.

[52] Zwaan R.A. (1996). Processing Narrative Time Shifts. J. Exp. Psychol. Learn. Mem. Cogn..

[53] Lorch R.F., Lorch E.P., Klusewitz M.A. (1993). College Students’ Conditional Knowledge about Reading. J. Educ. Psychol..

[54] Van Dijk T.A. (1980). The Semantics and Pragmatics of Functional Coherence in Discourse. Speech Act Theory Ten Years Later.

[55] Frazier L., Rayner K. (1982). Making and Correcting Errors during Sentence Comprehension: Eye Movements in the Analysis of Structurally Ambiguous Sentences. Cogn. Psychol..

[56] Meseguer E., Carreiras M., Clifton C. (2002). Overt Reanalysis Strategies and Eye Movements during the Reading of Mild Garden Path Sentences. Mem. Cogn..

[57] Hyönä J., Lorch R.F., Kaakinen J.K. (2002). Individual Differences in Reading to Summarize Expository Text: Evidence from Eye Fixation Patterns. J. Educ. Psychol..

[58] Jarodzka H., Brand-Gruwel S. (2017). Tracking the Reading Eye: Towards a Model of Real-world Reading. Comput. Assist. Learn..

[59] Schmitz A., Gräsel C., Rothstein B. (2017). Students’ Genre Expectations and the Effects of Text Cohesion on Reading Comprehension. Read. Writ..

[60] McDaniel M.A., Einstein G.O. (1989). Material-Appropriate Processing: A Contextualist Approach to Reading and Studying Strategies. Educ. Psychol. Rev..

[61] Breen M., Clifton C. (2011). Stress Matters: Effects of Anticipated Lexical Stress on Silent Reading. J. Mem. Lang..

[62] Beck J., Konieczny L. (2021). Rhythmic Subvocalization: An Eye-Tracking Study on Silent Poetry Reading. J. Eye Mov. Res..

[63] Koops Van ’T Jagt R., Hoeks J., Dorleijn G.J., Hendriks P. (2014). Look before You Leap: How Enjambment Affects the Processing of Poetry. Sci. Study Lit..

[64] Fechino M., Jacobs A.M., Lüdtke J. (2020). Following in Jakobson and Lévi-Strauss’ Footsteps: A Neurocognitive Poetics Investigation of Eye Movements during the Reading of Baudelaire’s ‘Les Chats’. J. Eye Mov. Res..

[65] Gernsbacher M.A. (1991). Cognitive Processes and Mechanisms in Language Comprehension: The Structure Building Framework. Psychology of Learning and Motivation.

[66] Gernsbacher M.A. (1997). Two Decades of Structure Building. Discourse Process..

[67] Hyönä J., Nurminen A. (2006). Do Adult Readers Know How They Read? Evidence from Eye Movement Patterns and Verbal Reports. Br. J. Psychol..

